# Testis-expressed profilins 3 and 4 show distinct functional characteristics and localize in the acroplaxome-manchette complex in spermatids

**DOI:** 10.1186/1471-2121-10-34

**Published:** 2009-05-06

**Authors:** Martina Behnen, Kai Murk, Petri Kursula, Heike Cappallo-Obermann, Martin Rothkegel, Abraham L Kierszenbaum, Christiane Kirchhoff

**Affiliations:** 1 Department of Andrology, University Hospital Hamburg-Eppendorf, Hamburg, Germany; 2 Zoological Institute, University of Braunschweig, Braunschweig, Germany; 3 Department of Biochemistry, University of Oulu, Oulu, Finland; 4 Department of Cell Biology and Anatomy, The City University of New York Medical School, New York, NY, USA

## Abstract

**Background:**

Multiple profilin isoforms exist in mammals; at least four are expressed in the mammalian testis. The testis-specific isoforms profilin-3 (PFN3) and profilin-4 (PFN4) may have specialized roles in spermatogenic cells which are distinct from known functions fulfilled by the "somatic" profilins, profilin-1 (PFN1) and profilin-2 (PFN2).

**Results:**

Ligand interactions and spatial distributions of PFN3 and PFN4 were compared by biochemical, molecular and immunological methods; PFN1 and PFN2 were employed as controls. β-actin, phosphoinositides, poly-L-proline and mDia3, but not VASP, were confirmed as *in vitro *interaction partners of PFN3. In parallel experiments, PFN4 bound to selected phosphoinositides but not to poly-L-proline, proline-rich proteins, or actin. Immunofluorescence microscopy of PFN3 and PFN4 revealed distinct subcellular locations in differentiating spermatids. Both were associated first with the acroplaxome and later with the transient manchette. Predicted 3D structures indicated that PFN3 has the actin-binding site conserved, but retains only approximately half of the common poly-L-proline binding site. PFN4, in comparison, has lost both, polyproline and actin binding sites completely, which is well in line with the experimental data.

**Conclusion:**

The testis-specific isoform PFN3 showed major hallmarks of the well characterized "somatic" profilin isoforms, albeit with distinct binding affinities. PFN4, on the other hand, did not interact with actin or polyproline *in vitro*. Rather, it seemed to be specialized for phospholipid binding, possibly providing cellular functions which are distinct from actin dynamics regulation.

## Background

Profilins are small, ≈ 14-kDa intracellular proteins which are crucial for actin microfilament dynamics ([1-4]; for review, see [5]). Their ubiquity, abundance, and necessity for life in higher organisms underscore their general importance ([6]; for review, see [7]). Despite their small size, their functions are amazingly diverse. Through binding to numerous protein ligands, profilins are components of complex protein networks (for review, see [8]). Interactions with components of the phosphatidylinositol cycle [9] and the rac-rho pathway [10,11] implicate them as a link through which the actin cytoskeleton communicates with the major signalling pathways of the cell. Accordingly, reducing the amount of profilin protein, *e.g*. by gene deletion, often has severe or even fatal consequences on the viability of the afflicted organism.

Profilins constitute a large and diverse protein family. Multiple isoforms exist in many species, being encoded by separate genes, or in some cases translated from mRNA splice variants. In animals and higher plants, isoforms may be expressed in a tissue-specific manner. Moreover, profilins are found at different subcellular locations (for review, see [7]). Enrichment at dynamic plasma membranes was confirmed for various cells types. Also, profilins were observed in association with internal membranes involved in vesicular transport [12]. Finally, profilins are constituents of the cell nucleus (for review, see [7] and [8]). Although the overall structure is conserved, sequence homologies between profilins from different species, and also between different isoforms from the same organism, are low ([13]; for review, see [8]). It was, nevertheless, reported that the overall functional properties of different profilins are similar, and that one isoform can be interchanged with another one from quite a distant source [14]. On the other hand, structural differences, which determine *in vitro *affinities for various ligands [15,16], preferential protein complex formation in different cell types, and different subcellular locations may be important clues of divergent, possibly non-overlapping *in vivo *functions of different isoforms.

Despite extensive studies, the significance of the multiple profilins, their tissue-specificity and distinct subcellular locations have remained enigmatic. At least four different profilins were demonstrated to be present in the mammalian testis [17-20], a complexity which was not observed in somatic tissues. The mammalian testis may, thus, serve as a model to question whether profilin isoforms may fulfil distinct functions. Profilin-1 (PFN1) is ubiquitous and essential for cell viability [6]; its expression in all cell types of the testis, including spermatogenic cells, thus seems obvious. In comparison, profilin-2 (PFN2) is predominantly found in the nervous system and has acquired more specialized functions in regulating neuronal activity [21]; it may represent a cell type-specific isoform also in the testis. While PFN1 and PFN2 were both demonstrated in the somatic Sertoli cells [19], a third isoform, profilin-3 (PFN3), is expressed solely in spermatids [18,20]. Most recently, profilin-4 (PFN4) was characterized as a novel isoform. It shows less than 30% amino acid identity with the other mammalian profilins; still, database searches produced significant alignments with the conserved profilin domain. PFN4 is also highly expressed during spermatogenesis, but is distinct from PFN3 in its temporal expression pattern [20].

Based on sequence comparisons, it was speculated that the testis-expressed PFN3 and PFN4 might have altered binding capacities for actin and proline-rich ligands [13]. However, such diverse characteristics remained to be experimentally shown. In the present study, using yeast two-hybrid interaction assays and various biochemical methods, the binding capacities of PFN3 and PFN4 for proline-rich ligands, actin, and phosphoinositides were studied in comparison with PFN1 and PFN2. Subcellular locations in differentiating spermatids were studied by immunofluorescence. Three-dimensional structural models were also built to explain the functional properties of PFN3 and PFN4.

## Results and Discussion

### PFN3 and PFN4 have different *in vitro *affinities for protein ligands

Actin monomers, poly-L-proline (PLP), and proline-rich proteins have been confirmed as *in vitro *ligands for each profilin isoform tested so far. In addition, *in vivo *interactions of various profilins have been verified with actin (for review, see [3]), and proline-rich proteins, including the vasodilator-stimulated phosphoprotein (VASP; [22]) and the mammalian homologues of Drosophila diaphanous (mDia), members of the formin gene family [23]. In analogy with this, several databases of known and predicted protein-protein interactions suggested that the testis-expressed isoforms PFN3 and PFN4 would likewise associate with these ligands [24,25]. We asked whether PFN3 and PFN4 indeed shared these affinities.

**Figure 1:**
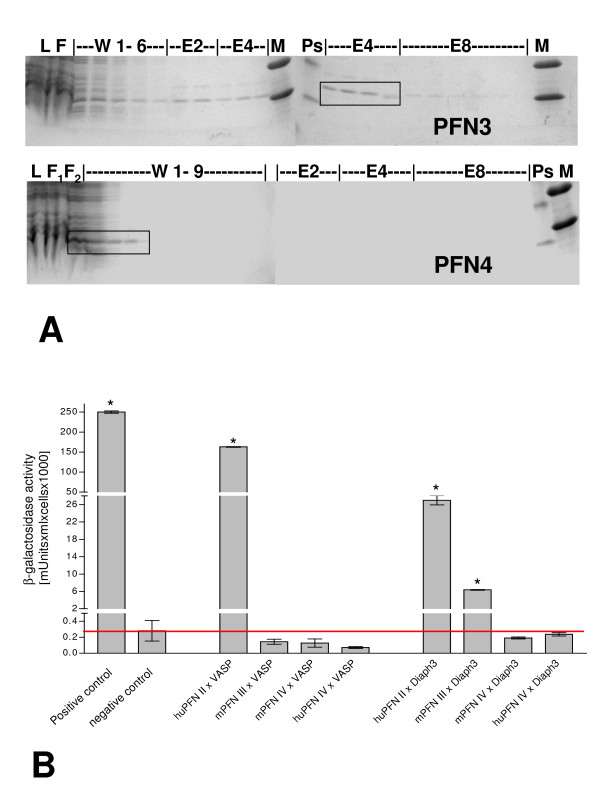
**Poly-proline interaction of PFN3 and PFN4**. (A) Poly-L-proline (PLP) affinity chromatography. PLP interaction of PFN3 (upper panel) and PFN4 (lower panel) was examined by column chromatography and fractions analysed on SDS gels of which only the 14–20 kDa regions are shown. PFN3 bound to the PLP-column and was eluted by 2 M and 4 M urea (protein bands highlightened by frame), while PFN4 failed to interact with PLP and was washed from the column during initial washing steps (protein bands highlightened by frame). L: cell lysate before column; F: flow-through, W: wash without urea (1–9 indicate fraction numbers); E2: 2 M urea eluate; E4: 4 M urea eluate; E8: 8 M urea eluate; M: low mass ladder, Ps: pre-stained marker. (B) Quantitative β-galactosidase assay for selective pair wise Y2H interaction of PFN2, PFN3, and PFN4 with polyproline-rich VASP and DIAPH3. Diploids containing PFN2 and VASP or PFN2 and Diaph3 showed significant activity, reflecting the ability of PFN2 interact with these proteins. PFN3-containing diploids revealed significant but weaker binding to DIAPH3, and no binding to VASP. PFN4 failed to interact with both proteins. Bars show quantitative β-galactosidase activity [milliunits/(ml × cells)] of colonies grown in SD/-LTHA (Leu- Trp- His- Adenine-) high stringency medium. The red line marks the level of the negative control. * designates significant β-galactosidase activities. Positive control: diploids from SD/-LTHA medium containing p53 (pGBKT7–53) and SV40 large T-antigen (pGADT7-T). Negative control: diploids with HE6/GPR64-C-terminus (pGBKT7-H21-21-1) und SV40 T-antigen (pGADT7-T) showing no interaction.

Protein lysates containing pET-expressed ("untagged") PFN3 and PFN4 proteins were employed in PLP affinity chromatography. PFN3 bound to the PLP affinity column (Figure 1A, upper panel); however, compared to PFN1 and PFN2, the association was weaker, since PFN3 was eluted from the PLP column at 2 to 4 M urea (compare PLP affinity chromatography data presented in [26]). Under the same experimental conditions, PFN4 failed to interact with PLP (Figure 1A, lower panel), suggesting that different from the above predictions proline-rich protein ligands may not be targets for this isoform. Since PLP affinity chromatography is routinely employed to purify native as well as recombinant profilins, the lack of polyproline interaction caused complications in the purification of untagged PFN4.

Selective pair wise tests for yeast two-hybrid (Y2H) interaction ("minimatings"), assaying potential differences of PFN3 and PFN4 in their affinities for specific proline-rich proteins, i.e. VASP and mDia3 (Figure 1B), were performed as an independent method. PFN2a, which is known to bind VASP and p140mDia [15,22,23], was included as a positive control. Expression of the LacZ reporter was used as a more quantitative indicator for the strength of protein-protein interactions. mDia3, but not VASP, was confirmed as an *in vitro *binding partner of PFN3 (Fig. 1B). The interaction of mDia3 with PFN3 was weaker compared to PFN2a; parallel Y2H assays employing PFN4, in comparison, were negative (Figure 1B), confirming the results of the PLP affinity chromatography. Notably, our previous microarray analyses of human testis tissue ([27] and own unpublished results) had shown that at least five mDia3-encoding mRNAs variants, including the one employed in the Y2H assays here, were highly expressed in the testis and were most abundant in tissue samples, which contained post-meiotic germ cells. Thus, mDia3 proteins may still represent *in vivo *interaction partners of PFN3.

**Figure 2:**
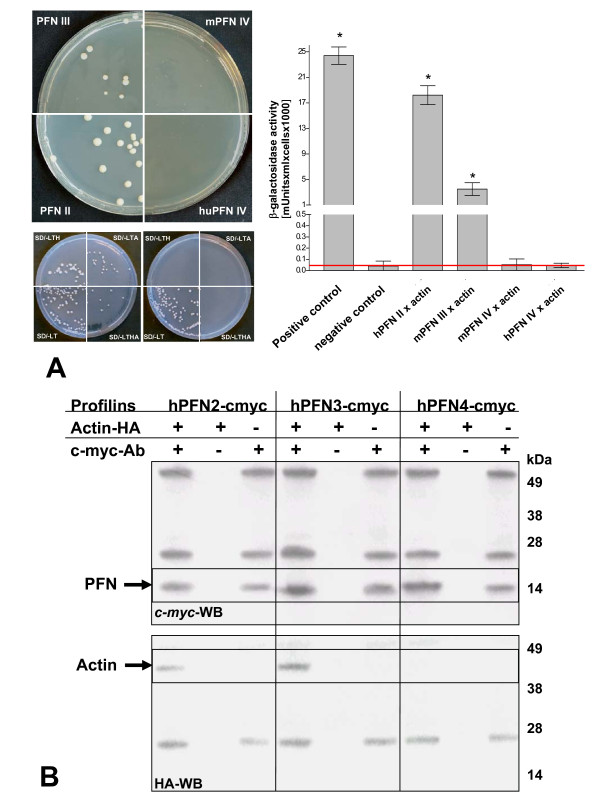
***In vitro *interaction of PFN3 and PFN4 with actin**. A) Yeast two-hybrid (Y2H) interaction assays of PFN2, PFN3, and PFN4 with β-actin (left panel). Plate growth assays were performed on minimal media in the absence of histidine (SD/-LTH) or adenine (SD/-LTA) or both (SD/-LTHA). Interaction is indicated by growth of diploid colonies. Upper panel shows assay for profilin-pGADT7 × actin-pGBKT7 on SD/-LTHA medium. Lower panel shows positive control (actin-pGBKT7 × huProfilin-2-pGADT7) on the left;, negative control (actin-pGBKT7 × pGADT7) on the right. Quantification was carried out by β-galactosidase assay (right panel) using diploids from SD/-LTHA-medium Red line marks the negative value; * designates significant activities. Positive control: diploids containing p53 (pGBKT7–53) and SV40 large T-antigen (pGADT7-T). Negative control: diploids with HE6/GPR64-C-terminus (pGBKT7-H21-21-1) und SV40 T-antigen (pGADT7-T). B) Western blot analysis of hemagglutinin epitope (HA)-tagged β-actin co-immunoprecipitated with c-myc-tagged PFN2, PFN3, and PFN4. Analyses employing the c-myc- antibody showed that the three profilin isoforms (~14 kDa) were precipitated (upper panel; highlightened by arrow). Analyses employing the anti-HA- antibody showed that actin-HA (~45 kDa) was co-precipitated with PFN2 and PFN3, but not with PFN4 (bottom panel; highlightened by arrow). The ~28 kDa and ~50 kDa protein bands resulted from the light and heavy chains of the c-myc-antibody.

Binding of the testis-specific profilin isoforms to β-actin was similarly tested by a pair wise Y2H interaction, confirming PFN3 as an actin-binding isoform (Figure 2A). Parallel assays employing PFN4 were all negative (Figure 2A), suggesting that neither proline-rich proteins nor actin were targets of this unusual isoform. The failure of PFN4 to interact with actin contrasts with all other profilins studied thus far, most of which bind actin monomers with micromolar affinity. The Y2H method, however, is prone to false-negative discovery. By attaching domains from a transcription factor to the bait and prey proteins, true interactions may be missed if fusions place these attachments at important interacting actin and/or profilin interfaces [28]. To confirm the Y2H results by two independent methods, co-immunoprecipitation of β-actin and actin polymerization assays were performed. For co-immunoprecipitation PFN2, PFN3, and PFN4 were translated *in vitro *as N-terminal fusions to a c-myc-tag; a c-myc-antibody was employed to immunoprecipitate the profilins in the presence of HA-tagged β-actin. While PFN2 and PFN3 effectively co-precipitated with β-actin as evidenced by their retention by protein A beads, PFN4 failed to associate with β-actin also under these experimental conditions (Figure 2B). In a control experiment, in the absence of profilins, β-actin was not precipitated with protein A beads (data not shown).

**Figure 3:**
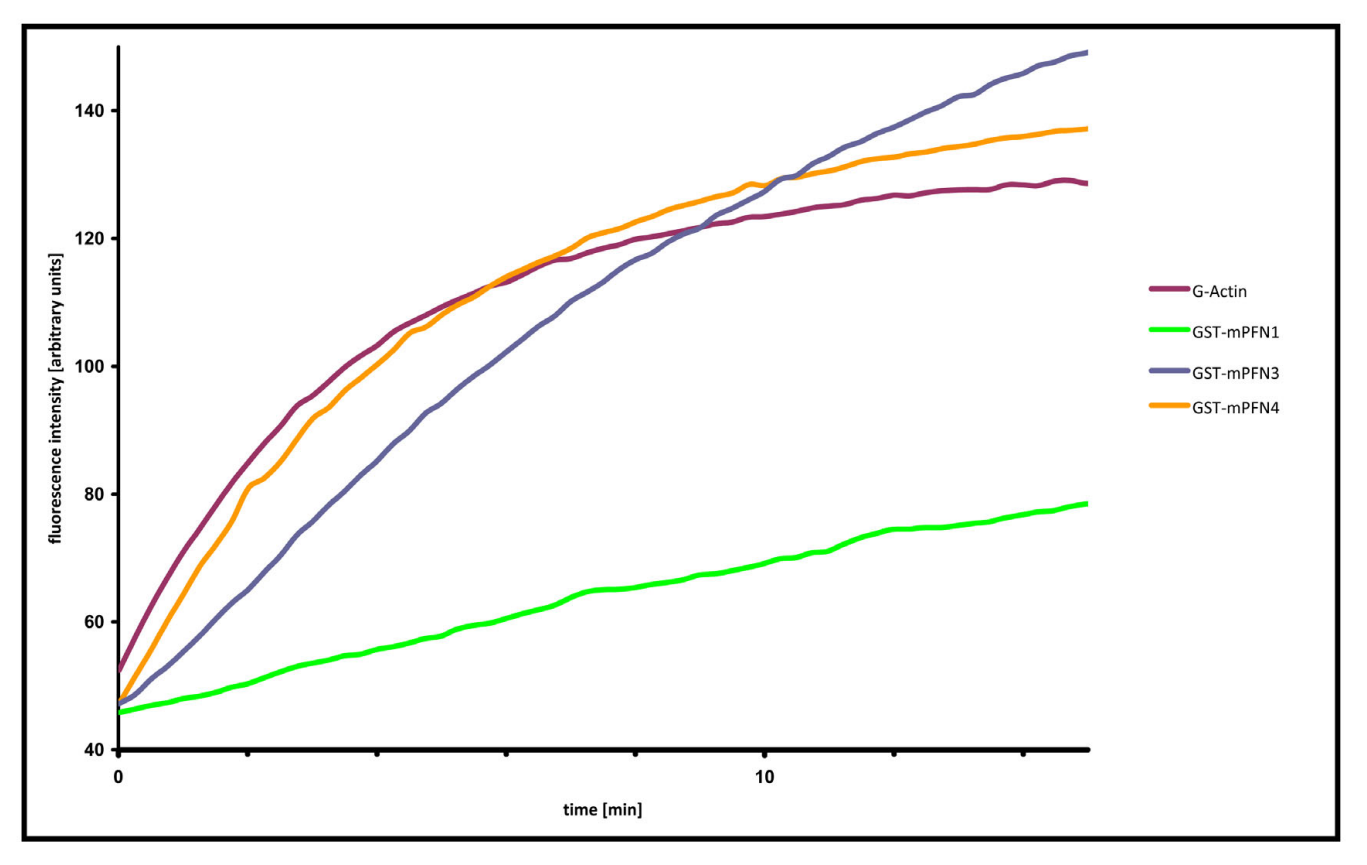
**Effect of GST-tagged profilin isoforms on β-actin polymerization kinetics**. 5 μM actin and 15 μM each of GST-PFN1, GST-PFN3, and GST-PFN4 were pre-incubated prior to induction of actin polymerization by addition of KCl and MgCl_2_. Time courses of actin alone (red) or in the presence of GST-PFN1 (green), GST-PFN3 (blue) or GST-PFN4 (orange) are shown. PFN1 markedly delayed actin polymerization, PFN3 influenced polymerization kinetics to a lower extent, and PFN4 had no obvious effect.

We probed the influence of recombinantly expressed GST-fusions of PFN3 and PFN4 on salt-induced actin polymerization using the pyrenyl fluorescence assay [29]. Labelled skeletal muscle G-actin was incubated with or without the purified GST-fused profilins (Figure 3). GST-PFN1, which served as a control, showed a significant retardation of actin polymerization. Under the same experimental conditions, PFN3 also markedly reduced polymerization kinetics, albeit to a lesser extent. PFN4, on the other hand, did not influence the actin polymerization kinetics at all (Figure 3). In summary, concerning PFN3 protein interaction partners, our results confirmed and extended the results by Hu et al. [17], suggesting that PFN3 showed major hallmarks of the well characterized profilins. PFN4, on the other hand, did not bind the tested protein ligands *in vitro *and thus may have *in vivo *functions different from regulating actin dynamics. It should be noted that spermatids contain several actin-related proteins [30]. Recently, the actin-related protein ArpM1 was identified as an interaction partner of PFN3 [31]. The interaction of PFN4 and actin-related proteins expressed in spermatids deserves further evaluation.

### PFN3 and PFN4 differentially interact with phospholipids

Profilins interact with anionic phospholipids ([32]; for review, see [8]). Individual phospholipids show distinct subcellular distributions and perform distinct biological roles (for review, see [33]), with steady-state concentrations of PtdIns(4,5)P_2 _predominating at plasma membranes, PtdIns(3)P on endosomes, and PtdIns(4)P on the Golgi. Thus, through differential binding to these phospholipids *in vivo*, profilin isoforms may be selectively directed to functionally appropriate subcellular sites. Selective interaction with phosphoinositides was indeed the first functional difference reported between different profilin isoforms [34,35]. Moreover, it was shown that binding to PtdIns(4,5)P_2 _may effectively compete for PLP interaction of profilin 1 [36]. More recently, neighbouring binding sites for these competing ligands have emerged which may explain the competing interactions [37,38].

**Figure 4:**
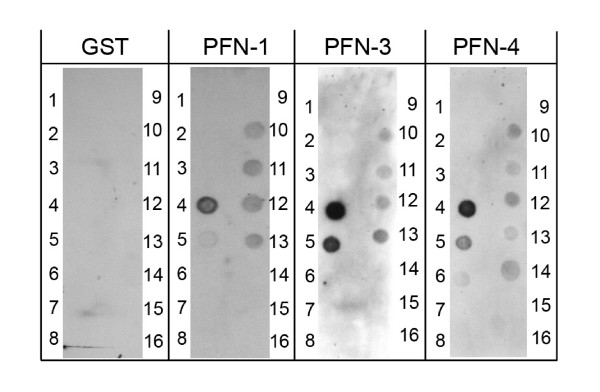
**Protein-lipid overlay assay of profilins**. Phosphoinositide (PIP) overlay assay of Glutathione-S-transferase (GST)-fusion proteins showed selective binding of GST-PFN1, GST-PFN2, and GST-PFN4 to nitrocellulose-bound phosphoinositides (100 picomoles per spot). GST alone did not bind. PIP strips™ were incubated with 0.5 μg/ml of each fusion protein as indicated. Layout of strips (according to Molecular Probes product information) was as follows. Spot # 1: Lysophosphatidic acid; # 2: Lysophosphatidylcholine; # 3: Phosphatidylinositol (PtdIns); # 4: PtdIns(3)P; # 5: PtdIns(4)P; # 6: PtdIns(5)P; # 7: Phosphatidylethanolamine; # 8: Phosphatidylcholine; # 9: Sphingosine 1-phosphate; # 10: PtdIns(3,4)P2; # 11: PtdIns(3,5)P2; # 12: PtdIns(4,5)P2; #13: PtdIns(3,4,5)P3; #14: Phosphatidic acid; #15: Phosphatidylserine; #16: Blank.

Phospholipid interactions of GST-fusion proteins of PFN1, PFN3, and PFN4, respectively, were assayed by protein-lipid overlay [39]. Each of the recombinant fusions strongly bound to PtdIns(3)P, and to a lesser extent to its phosphorylated products and PtdIns(4,5)P_2 _(Figure 4). Unlike GST-PFN1 (and also other profilins), GST-PFN3 and GST-PFN4 both showed *in vitro *affinity towards PtdIns(4)P, suggesting that *in vivo*, in addition to endosomes, PFN3 and PFN4 may be associated with the Golgi apparatus. For GST-PFN4, a weak but reproducible binding to phosphatidic acid (PA) was additionally observed (Figure 4). PA is an abundant component (1–4%) of most cellular membranes. It is also a signalling lipid, since its regulated formation can constitute an important signal for many downstream responses, including actin polymerization in spermatozoa [40]. In addition to this role, PA appears to regulate enzymes in phospholipase D pathways directly. The list of PA-binding proteins is rapidly expanding (for review, see [41]); however, PA-binding of profilins has not been previously reported. The specificity of the observed interaction must be considered critically as the isoelectric point of PFN4 is basic (pI 8.8); still, GST-PFN3 did not bind PA under the same experimental conditions, despite of the highly basic character of PFN3 (pI 9.5).

### PFN3 and PFN4 localize in the acroplaxome-manchette complex in spermatids

**Figure 5:**
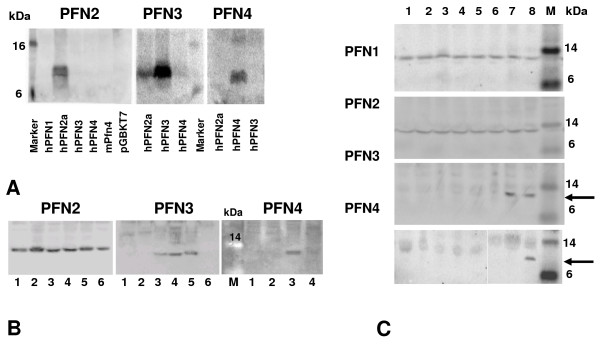
**Specificity of anti-profilin antibodies**. A) Western blot analysis of *in vitro *translated profilins employing antibodies directed against PFN2 (left panel), PFN3 (middle panel), and PFN4 (right panel). Note that there is no cross-reactivity of anti-PFN3 and anti-PFN4 antibodies. B) Western blot analysis of PFN2 (left panel), PFN3 (middle panel), and PFN-4 (right panel) protein expression in human testes showing varying degrees of spermatogenetic failure. Lane 1: Sertoli-cell-only appearance; lane 2: maturation arrest at meiosis; lane 3: hypospermatogenesis; lane 4: full spermatogenesis; lane 5: full spermatogenesis; lane 6: salivary gland as control. Note that PFN3 and PFN4 immunoreactive protein bands of ≈ 14 kDa were solely observed in tissue samples containing sufficient amounts of spermatids while PFN2 was detected in each tissue analysed. C) Western blot analysis of PFN1 and PFN2 (upper panels), PFN3 (middle panel), and PFN4 (lower panel) proteins in rat testis at various stages of postnatal development. Lane 1: day-15; lane 2: day-18; lane 3: day-20; lane 4: day-24; lane 5: day-26; lane 6: day-28; lane 7: day-30; lane 8: day-45; lane 9: day-60 testes. Note that PFN3 and PFN4 immunoreactive protein bands of ≈ 14 kDa were solely observed in testes at developmental stages, which contained sufficient amounts of elongating spermatids (high lightened by arrows). PFN1 and PFN2 immunoreactive bands, in comparison, were observed at all stages analysed.

Cross-reactivity of the profilin antisera employed in immunostaining procedures was initially studied by Western blot analysis using *in vitro *translated profilins (Figure 5A). Antisera generated against PFN3 and PFN4 did not cross-react; weak cross-reactivity of the anti-PFN3 antiserum was observed only with *in vitro*-translated PFN2. In protein extracts of human adult testis tissue (Figure 5B), and also in rat testis samples collected during the first wave of rat spermatogenesis (Figure 5C), anti-PFN3 and anti-PFN4 immunoreactive bands of ≈ 14-kDa were seen only in those tissue samples which contained spermatids, confirming at the protein level the previously reported stage- and cell-type specific expression patterns of PFN3 and PFN4 [20]. In comparison, anti-PFN1 and PFN2 immunoreactive bands were obvious in each testis sample analysed (Figure 5C).

Our previous studies suggested that PFN3 and PFN4 accumulated near the acrosome-acroplaxome-manchette complex of differentiating spermatids ([2]0 and data not shown). The acroplaxome is an F-actin/keratin 5-containing cytoskeletal plate, which anchors the acrosome to the spermatid nucleus [42]. Pro-acrosomal vesicles derived from the Golgi apparatus [43] are transported by F-actin- and microtubule-based molecular motors and dock and fuse along the acroplaxome (for review, see [44]). The manchette is a transient structure developed subjacent to the marginal ring of the acroplaxome. It consists of a perinuclear ring with inserted microtubules as well as associated F-actin. The manchette participates in the transport of cargoes to the developing spermatid tail and in nucleo-cytoplasmic trafficking during spermatid head shaping [45].

**Figure 6:**
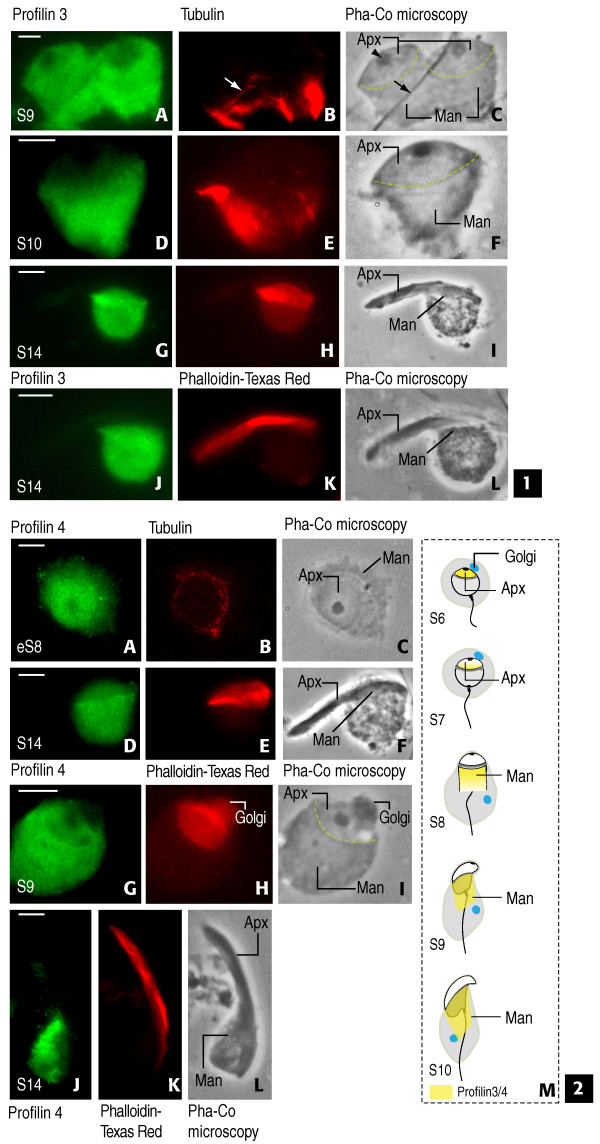
**Localization of profilin 3 (panels 1) and profilin 4 (panels 2) during rat spermiogenesis**. 1, panels A-C: S9 spermatids display profilin 3 immunoreactivity (A) in acroplaxome (F-actin component stained with phalloidin-Texas Red) and manchette (detected with tubulin monoclonal antibody). Manchette is caudal to acroplaxome (B). Arrows in B and C indicate tubulin immunoreactive spermatid tail. Arrowhead in C (Pha-Co microscopy: phase-contrast microscopy) points to acrosomal granule. Apx: acroplaxome. Man: manchette. Panels D-F: S10 spermatid. Profilin 3 is predominant in manchette. Panels G-I: S14 spermatid. Profilin 3 extends into cytoplasm caudal to manchette. Panels J-L: S14 spermatid. Panel K illustrates position of acroplaxome. Note that profilin 3 is not associated with the acroplaxome but with the manchette. 2, panels A-C. Early S8 (eS8). Profilin 4 is predominant in acroplaxome (A) at the initiation of the manchette (B). C indicates positions of acroplaxome (Apx) and manchette (Man). Panels D-F. S14 spermatid. Profilin 4 is seen in manchette but not in acroplaxome. Panels G-I. S9 spermatid. Profilin 4 is predominant in manchette (G). Position of acroplaxome is shown in H. Note that adjacent Golgi is slightly stained. I indicates acroplaxome (Apx), manchette (Man) and Golgi resolved by phase-contrast. Panels J-L. S14 spermatid. Profilin 4 is restricted to manchette (J) and not seen in acroplaxome (seen in K). L indicates manchette (Man) and acroplaxome (Apx). Panel M provides diagrammatic summary (not to scale) of profilin 3 and profilin 4 localization sites during S6 to S10. The acroplaxome is immunoreactive in S6 spermatids. Acrosomal immunoreactivity decreases in apical region in S7 spermatids preceding the onset of manchette assembly (S8). Profilin 3 and profilin 4 are predominant in the manchette of S9 and S10 spermatids. Note migration of Golgi from apical to caudal position in spermatid cytoplasm. Scale bars: 2 μm.

Indirect immunofluorescence of spermatids isolated from spermatogenic stage-specific rat seminiferous tubules was used to determine the localization of PFN3 and PFN4 throughout spermiogenesis. Phalloidin-Texas Red was used to monitor the F-actin-containing acroplaxome; tubulin monoclonal antibody was used to determine the position of the manchette with respect to the acroplaxome. PFN3 immunoreactivity was mainly observed in the acroplaxome of round spermatids and may be correlated with the presence of F-actin (Figure 6/1, panels A-L). During the progression of spermiogenesis, PFN3 acroplaxome immunoreactivity gradually disappeared and became apparent in the developing manchette of spermatids step 8 (S8) (Figure 6/1, panels D-F). Coinciding with the initiation of manchette disassembly in S14 spermatids, PFN3 immunoreactivity was seen in the cytoplasm subjacent to the disassembling manchette (Figure 6/1, panels G-I). At this stage, the phalloidin-stained acroplaxome was devoid of PFN3 (Figure 6/1, panels J-L). A similar immunoreactive staining pattern was observed with anti-PFN4 serum in rat (Figure 6/2, panels A-L) and also in human spermatids (Additional File 1). In agreement with the immunoblotting data, normal rabbit IgG controls did not generate a fluorescence signal (data not shown). Panel M in Figure 6/2 summarizes diagrammatically the localization of PFN3 and PFN4, first in the acroplaxome and later in the manchette during spermatid head development. These observations suggested that, despite their differing functional characteristics *in vitro*, the location sites of PFN3 and PFN4 proteins in spermatids were similar. *In vivo *functions of the tissue- and cell type-restricted isoforms, however, remain obscure. Transgenic mouse models may help to clarify whether and how PFN3 and PFN4 impact on acrosome formation and/or spermatid head shaping.

### Structural models explain the observed biochemical properties of PFN3 and PFN4

**Figure 7:**
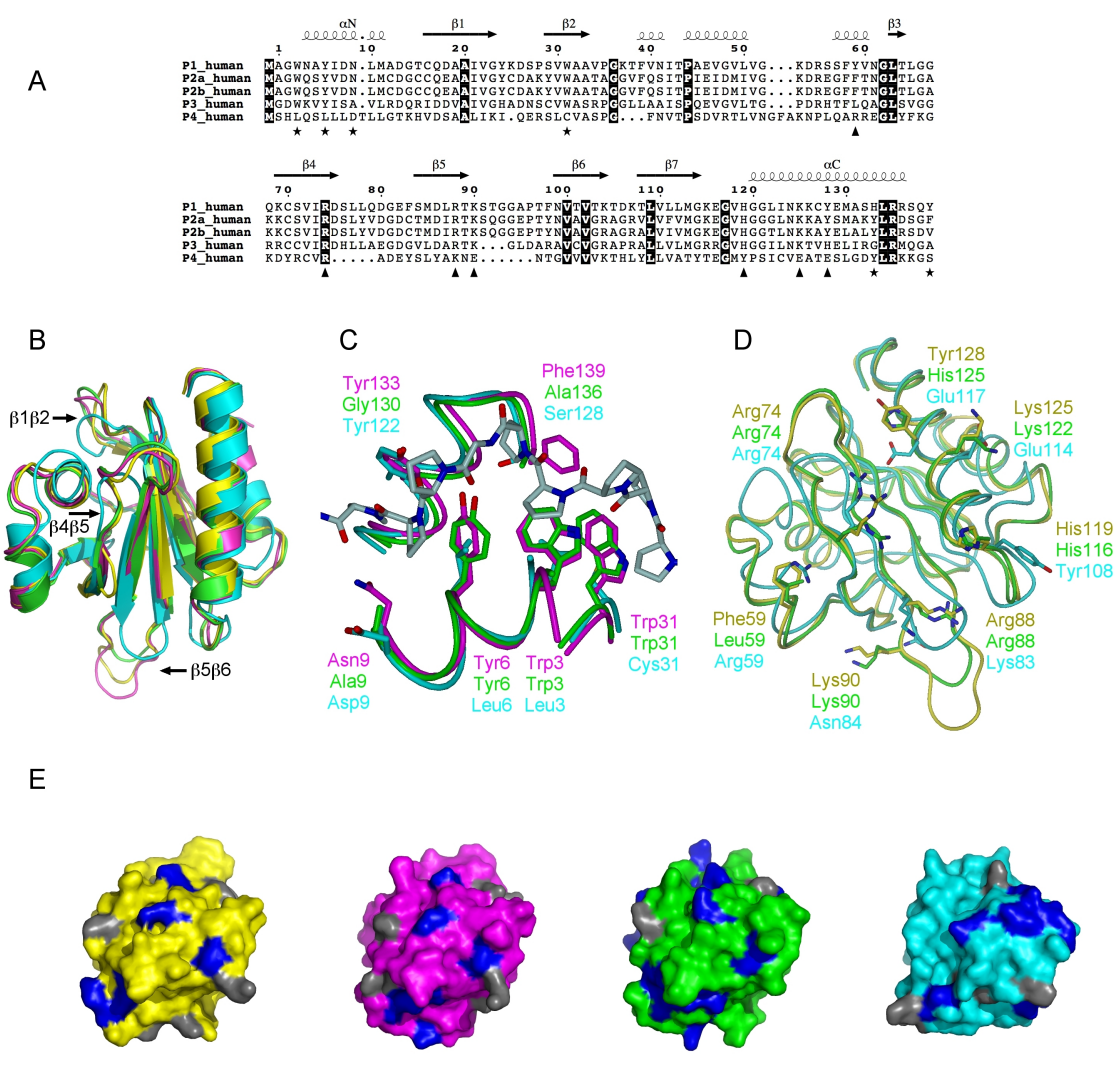
**Three-dimensional models of PFN3 and PFN4**. A. Sequence alignment between human profilins. Fully conserved residues are on dark background. Key residues of PLP binding are indicated by asterisks, those involved in binding actin with triangles. Secondary structures are derived from bovine PFN1 crystal structure. For compatibility with most publications, the first methionine is not considered in sequence numbering. B. Superposition of bovine PFN1 (yellow), mouse PFN2a (magenta), human PFN3 (green), and human PFN4 (cyan). PFN1 structure is from the profilactin complex (PDB core 1HLU) and PFN2a from the complex with a VASP peptide (PDB code 2V8C). Loops variable in length in PFN3 and/or PFN4 are marked. C. Comparison of the PLP-binding sites of PFN2a, PFN3, and PFN4. Shown is also the PFN2a-VASP complex, colouring as in 6B. Only half of the binding site is conserved in PFN3, no conservation is seen in PFN4. Key residues for peptide binding are indicated. D. Actin-binding sites of PFN1, PFN3, and PFN4, colouring as in 6B. For clarity, actin is not shown; view is from the direction of actin onto the actin-binding surface on profilin, side chains of key profilin residues are shown. E. Comparison of PtdIns(4,5)P2 binding surfaces of PFN1, 2a, 3, and 4 (left to right, respectively). Profilins are coloured as in 6A, and all arginine residues, crucial for PtdIns(4,5)P2 binding, are highlighted in blue; lysine residues are shown in gray.

In order to gain a deeper understanding of the observed functional differences, homology models were built for human PFN3 and PFN4, and compared to the known structures of human PFN1 and PFN2a. The sequence alignments of all known human profilins are shown in Figure 7A, and those used for modelling in additional file 2. Both PFN3 and PFN4 are predicted to be folded like other profilins, with a central 7-stranded antiparallel beta-sheet covered on both faces by two alpha helices. The PFN4 sequence, however, is ten amino acids shorter which is structurally reflected by three loops, *i.e*. β1β2, β4β5, and β5β6, being shorter in the PFN4 protein (Figure 7B). One of the loops, β5β6, is also shorter in PFN3.

#### The poly-L-proline binding site

The PLP binding site of profilins is formed by conserved aromatic amino acids located in the N- and C-terminal helices, such that the proline-rich peptide will bind to a groove between the two helices, interacting closely with the aromatic residues *via *hydrogen bonds and CH-pi interactions [15,38,46]. For example, five conserved aromatic residues (Trp3, Tyr6, Trp31, Tyr133, and Phe139) and also Asn9 are responsible for the PLP interaction of PFN2a (Figure 6C), and PFN1 is highly similar. The two aromatic residues on the C-terminal helix (Tyr133 and Phe139 in PFN2a) are not present in PFN3, which could lead to an altered specificity and/or lower affinity towards proline-rich sequences, as also evidenced by our binding assay. In PFN4, of the abovementioned aromatic residues, only Tyr122 is conserved (corresponding to His133 of PFN1 and Tyr133 of PFN2a). Thus, in this isoform, the aromatic surface of other profilins necessary for PLP binding is not present at all, which is in line with its observed inability to bind proline-rich sequences. While the overall sequence of PFN4 is more similar to the profilins of lower eukaryotes than to the other human/mammalian profilins [13], PFN4 seems to be the only family member so far, in which the entire PLP binding site is lacking, suggesting that it may have other binding partners instead.

#### The actin-binding site

In PFN1, residues centrally involved in actin binding include Phe59, His119, Arg74, Arg88, Lys90, Lys125, and Tyr128 (Figure 7D; [47]); these are fully conserved in PFN2a. In PFN3, this site is conserved, with some rather conservative amino acid sequence differences, such as the replacement of Phe59 and Tyr128 by Leu59 and His125, respectively, compared to PFN2a. These structural differences may have lowered or altered the affinity of PFN3 towards actin to some degree, when compared to PFN1 and PFN2a. In the PFN4 sequences, in contrast, there is no conservation of the known actin-binding residues of other profilins, which is well in line with our experimental data described above.

#### The *PtdIns*(4,5)*P*_2 _binding site

Based on mutagenesis studies and the localisation of sulphate and phosphate ions in crystal structures, the binding site for PtdIns(4,5)P_2 _has been approximately mapped in human PFN1 [37]. It involves several conserved basic amino acid residues on the profilin surface, generating a large binding surface of positively charged residues. In PFN2a, residues likely to contribute to PtdIns(4,5)P_2 _binding include arginines 74, 88, 104, 107, and 135, and these binding determinants are largely conserved in PFN1. Interestingly, Arg74 and Arg135 are among the few residues, which are conserved throughout the known human profilin isoforms, including PFN4 (Figure 7A). In PFN3, the putative common PtdIns(4,5)P_2 _binding site is well conserved. In PFN4, many basic residues are present as well, but the putative phospholipid binding site seems to have shifted (Figure 7E). This structural difference may explain the distinct phospholipid binding characteristics of PFN4 (see above).

## Conclusion

We report functional differences between the testis-expressed profilins PFN3 and PFN4 as revealed by *in vitro *assays. At the same time, structural homology models were built for human PFN3 and PFN4, which explained their different functional characteristics. By various methods, mDia3, but not VASP, was identified as a novel *in vitro *binding partner of PFN3, while PFN4 did not bind PLP or proline-rich proteins. So far, PFN4 seems to be the only profilin family member in which the entire PLP binding site is lacking. Moreover, different from PFN3, PFN4 did not interact with actin in three different *in vitro *assays deviating from all other profilins analysed thus far. Actually, the structure of PFN4 does not retain the actin-binding site of other profilins. A much lower affinity for actin or actin-related proteins is thus conceivable, suggesting that PFN4 may perform *in vivo *functions, which are distinct from regulating actin dynamics. PFN3 and PFN4 were both capable of *in vitro *phosphoinositide interaction, but differed in their selectivity towards specific phospholipids. In PFN3, the putative common PtdIns(4,5)P_2 _binding site of profilins is conserved while it seems to have shifted in PFN4. PFN3 and PFN4 coexist in the acroplaxome and the manchette of spermatids in a sequential developmental manner. They vanish gradually from the acroplaxome when the manchette fully assembles and spermatid head shaping is in progress. Although the presence of PFN3 can be correlated with the presence of F-actin and actin-related proteins in the acroplaxome, and to a lesser extent in the manchette, the visualization of PFN4 in the acroplaxome-manchette complex is intriguing in light of our finding that it does not interact with actin *in vitro*. An F-actin independent role of PFN4 may suggest novel functions related to the process of spermatid head shaping, and may have significant clinical implications in understanding idiopathic causes of male infertility associated with abnormal sperm head shaping.

## Methods

### Tissues and spermatogenic cells

Human testis tissue was obtained surgically from patients undergoing a testicular sperm extraction procedure (courtesy of Professor Dr. med. Wolfgang Schulze, Department of Andrology, University Hospital Hamburg-Eppendorf, Germany). Informed consent and Ethic Committee Approval was obtained (OB/X/2000), and the ethical principles for research involving human tissues as stated in the 52^nd ^World Medical Association *Declaration of Helsinki *were strictly observed. For protein extraction, tissue samples were submerged immediately in a cryoprotectant and snap-frozen in liquid nitrogen.

All animal housing and operation practices were in compliance with German Animal Welfare laws, and the *Guiding Principles in the Care und Use of Laboratory Animals *(DHEW Publication, NIH, 80-23) were observed in all cases. For protein extraction, male Wistar rats were obtained from the UKE animal house. Animals were sacrificed at days 10, 15, 20, 22, 26, 28, 30, 45, and 60 (n = 10 per age group) by decapitation (5- and 10-days-old animals) or CO_2 _inhalation (all others). Control tissues were taken from 60-days-old animals. Tissues were snap-frozen in liquid nitrogen immediately after removal. For the preparation of germ cells, adult male Sprague Dawley-rats (n = 3) were obtained from Charles River Inc. Animals were killed by CO_2_-asphyxiation, the testes removed, transferred to 32°C PBS solution and decapsulated.

### Antibodies

Polyclonal rabbit antisera against synthetic peptides were generated as described [20]. 14- and 15-mer linear oligopeptides from PFN2 and PFN3, i.e. CAYSMAKYLRDSGF and CEVGVLTGPDRHTFL, respectively, were used as antigen (Bioscience, Göttingen, Germany; Pineda-Antibodies Service, Berlin, Germany). Other antibodies used included a rabbit polyclonal anti-PFN1 (Novus Biologicals, Littleton, USA), rabbit anti-PFN4 peptide antiserum [20], monoclonal anti-α-tubulin DM1A (Sigma, Munich, Germany), monoclonal anti- actin JLA20 (Calbiochem, Schwalbach, Germany), rabbit anti-VASP [22], goat anti-GST (GE Healthcare, Freiburg, Germany) and monoclonal anti-c-myc (BD Biosciences, Heidelberg, Germany). Peroxidase-conjugated secondary antibodies were AffiniPure Fc-fragment goat anti-rabbit IgG and AffiniPure rabbit anti-mouse IgG (H+L), respectively (both from Jackson ImmunoResearch, Newmarket, UK). Antibodies used for immunocytochemistry are indicated below.

### Protein extraction

Tissue samples were homogenized three times for 10 s with the UltraTurrax (Art Labortechnik, Mühlheim) in 10 mM Tris, 1 mM EDTA, 0.5 mM DTT, 50 mM NaCl, 0.4% NP-40, 0.2% NaDOC, 0.04% SDS, Protease-inhibitor-cocktail (complete mini EDTA-free, Roche), pH 7.8 (100 μl solubilisation buffer per 30 mg tissue). After 30 min of solubilisation at 4°C on a rotating wheel, debris was removed by centrifugation (30 min, 13000 rpm, 4°C) and the supernatants collected. Protein concentrations were estimated employing the BioRad Protein-Assay according to the suggestions of the supplier (BioRad, München, Germany).

### *In vitro *translation and co-immunoprecipitation

C-myc-tagged profilins and HA-tagged β-actin were synthesized from pGBKT-7 and pGADT-7 plasmid constructs using the TNT^®^-T7 Quick Coupled Transcription, Translation System (Promega, Mannheim, Germany) according to manufacturer's instruction. 25 μl of β-actin *in vitro *translation reaction was co-incubated with 25 μl of profilin *in vitro *translation reaction at room temperature for 2 h. After incubation, 3 μl of anti c-myc-antibody (BD Biosciences) and 200 μl protein A-agarose (Roche Diagnostics, Penzberg, Germany) in PBS were added and the mixtures incubated overnight at 4°C. Protein A beads were then washed three times (10 s, 4°C, 7000 rpm) with 500 μl co-immunoprecipitation buffer containing 50 mM Tris (pH 8.0), 100 mM NaCl, 1 mM EDTA, 0.5% NP-40. Proteins were eluted at 95°C for 5 min in sample buffer (40 mM Tris, pH 6.8, 2% SDS, 100 mM DTT, 1 mM EDTA, 8% glycerol). After removal of protein A beads by centrifugation, 25 μl of protein supernatant were separated on a 4–12% gradient SDS-PAGE (see below), and profilins and actin visualized employing c-myc monoclonal antibody (BD Biosciences) and anti-HA-high affinity monoclonal antibody (clone 3F10, Sigma).

### Western blot analysis

Western blot analysis of *in vitro *translated proteins and protein extracts was carried out by standard procedures. Briefly, approximately 80 μg proteins per lane were separated on 4–12% NuPage^®^Novex Bis-Tris gradient gels (Invitrogen, Karlsruhe, Germany) and transferred to polyvinylidene difluoride membranes (Amersham) in a discontinuous buffer system using a semi-dry blotter. Immunodetection was carried out by blocking for 1 h in 1% Western-blocking reagent (Boehringer Mannheim, Germany) or in 5% ECL-blocking agent when using the ECL-Plus-system (GE Healthcare, Freiburg, Germany), followed by incubation with the first antibody over night at 4°C. Antibody dilutions were 1:1000 for anti-PFN1, 1:10000 for anti-PFN2, 1:700 for anti-PFN3 and 1:500 for anti-PFN4. Antibody binding was detected either by Cy5-conjugated AffiniPure goat anti-rabbit IgG or a peroxidase-conjugated AffiniPure Goat anti-rabbit IgG (both from Jackson Immuno Research, Newmarket, UK).

### Profilin expression in *E. coli *and PLP affinity chromatography

2xYT with kanamycin was inoculated 1:50 from a fresh ON culture of BL21(DE3)pLys cells harbouring the mouse PFN3 or -4 gene under control of the bacteriophage T7-promoter in the vector pET28a(+). At an OD_600 _of 0.5, protein expression was initiated by adding IPTG to a final concentration of 1 mM. After induction, the bacteria were grown for 4 h at 42°C. Then, the cells were pelleted by centrifugation (15 min at 6000 rpm, 4°C), resuspended in 25 ml of lysis buffer (50 mM Tris-HCl, 10 mM NaCl, 10 mM EDTA, 1.5% TritonX-100, 1:1000 Trasylol, 1 μM Pepstatin A, 50 μM Pefabloc SC) and incubated for 20 min on ice. After adding lysozyme, the solution was frozen overnight at -80°C. Next day, the solution was thawed at 37°C and then sonicated on ice 10 times 30 s at 80 W probe energy with 30-s intervals. The lysate was centrifuged at 4°C for 50 min at 14000 rpm. The supernatant was loaded onto a poly-L-proline column, washed and equilibrated with washing buffer (20 mM Tris-HCl, 150 mM NaCl). The column was washed with 5–10 column volumes of washing buffer and then with washing buffer including 2 M urea, to remove unbound protein. To analyze the binding affinity, profilins were then eluted with 4 M and 8 M elution buffer (4 M/8 M Urea in washing buffer). Fractions were collected and checked by standard SDS-PAGE.

### Yeast Two-Hybrid Interaction

Yeast strains AH109 and Y187 and pGBKT7 and pGADT7 plasmids were from the *Matchmaker Two-Hybrid System *3 (Clontech), providing HIS3, ADE2 and lacZ reporters and allowing high stringency assays. For bait and prey construction from human and mouse testes cDNAs, oligodeoxynucleotide primers as given in table 1 were employed in RT-PCR amplification and amplicons subcloned into the multiple cloning site of pGBKT7 and pGADT7 vectors. The coding region of the human VASP cDNA was likewise subcloned into pGADT7 and pGBKT7. The yeast strains were transformed with the constructs ([48]; *Quick and Easy Transformation Protocol*) and colonies grown according to the *Yeast Protocols Handbook *(Clontech, Heidelberg, 2001). Plasmid selection was maintained by growing cells in minimal medium (0.67% yeast nitrogen base, 2% glucose) supplemented with lysine, histidine, adenine and tryptophan (for pGADT7 selection) or leucine (for pGBKT7 selection). Mating tests were performed under conditions of increasing stringency according to the manufacturer's suggestions. Diploid colonies were replica-plated on minimal medium with high stringency and grown at 30°C for 4–8 days. Colonies were isolated and tested for the expression of the lacZ reporter using the *β-Galactosidase Assay Kit *(Pierce). Prey plasmids were isolated, transformed into *Escherichia coli*, and inserts verified by sequence analysis (MWG). Interactions were verified by plate growth assays on minimal mediums in the absence of histidine or adenine or both.

**Table 1:** Primers employed in RT-PCR

Primer	T_m_	product	Accession-No.
5'-CAGT***GAATTC***ATGGCCGGT TGGCAGAG-3' 5'-CGAT***GGATCC***AGCAGCTAGAACCCAGAGTC-3'	68°C 71°C	446 bp ORF (bp 99 → 526)	huProfilin2a NM_053024

5'-TT***GAATTC***ATGAGTGACTGGAAGGGCTACA-3' 5'-TT***GGATCC***GTTCACGGTTTATTCTGGTCTCC-3'	65°C 68°C	467 bp ORF (bp 59 → 524)	mProfilin3 NM_029303

5'-CAGT***GAATTC***AGCATGAGTCACTTGCG-3' 5'-GAT***GGATCC***TTAGTTTCCCTTTTTCCTTAG-3'	63°C 64°C	405 bp ORF (bp 861 → 1250)	mProfilin 4 NM_028376

5'-AGT***GAATTC***GGGAACATGAGCCATT-3' 5'-GAT***GGATCC***CTCTGATGACTTAACTTCCT-3'	61°C 65°C	406 bp ORF (bp171 → 557)	huProfilin4 BC029523

5'-TA***GAATTC***ATGGATGGATGATATCGCCGCGC-3' 5'-AA***GGATCC***AAGCCATGCCAATCTCA-3'	67°C 70°C	1236 bp ORF (bp74 → 1282)	hu β-actin, NM_001101

5'-CAGT***GAATTC***TTTACCGACCACCAAGAAACTCAG-3' 5'-CGAT***GGATCC***TCATATGGCACCCGAAAAGAGC-3'	68°C 71°C	668 bp, FH1 + part. FH2-domain (bp904 → 1551)	huDiaphanous3 NM_030932

### Actin polymerization assay

Muscle actin was purified from rabbit skeletal muscle as described [49] and labelled with pyrene according to Kouyama and Mihashi [29]. Recombinant profilins were expressed as glutathion-S transferase (GST) fusion proteins in *Escherichia coli *ER2566 (New England Biolabs, Heidelberg, Germany) and purified by glutathione sepharose affinity chromatography according to the manufacturer's instructions (GE Healthcare). Eluted profilins were dialysed against 20 mM Tris-Cl, pH 7.4, 0.2 mM CaCl_2_, 1 mM dithiothreitol and stored on ice. To determine their influence on actin polymerization, 5 μM α-actin (5% pyrene-labelled) in G-buffer (2 mM Tris-HCl, pH 7.5, 0.2 mM ATP, 0.1 mM CaCl_2_, 0.5 mM DTT) was incubated for 10 min at 20°C with or without 15 μM recombinant GST-fused profilins. Polymerization was initiated by the addition of MgCl_2 _and KCl (final concentration 1 mM and 50 mM, respectively). Fluorescence was monitored for 2 h at 366 nm excitation and 407 nm emission using a LS50B fluorimeter (Perkin Elmer, Langen, Germany).

### Protein-lipid overlay assay

PIP Strips™ membranes (Molecular Probes, Eugene, USA) were employed following the manufacturer's instructions. Briefly, after blocking with 3% bovine serum albumin (BSA) in TBS-T (10 mM Tris-Cl, ph 8.0, 150 mM NaCl, 0.1% (v/v) Tween 20) the lipid-containing membranes were incubated with 0.5 μg/ml GST fused profilins for 2.5 h. Membranes were washed three times with TBS-T + 3% BSA and the bound proteins detected by anti-GST antibody in conjunction with HRP-labelled secondary antibody and enhanced chemiluminescence.

### Immunofluorescence microscopy

Spermatogenic cells were collected from mechanically dissociated seminiferous tubular fragments (identified with a dissecting stereomicroscope as corresponding to stages I-XIV of rat spermatogenesis according to their transillumination pattern). Cells were placed in a drop of 3.7% paraformaldehyde (electron microscopy grade) in 0.1 M sucrose in phosphate buffer, pH 7.4, on microscope slides coated with Vectabond (Vector Laboratories, Burlingame, CA). This fixation procedure results in the preservation of the Golgi-acrosome-acroplaxome-manchette-nuclear relationship in spermatids, a condition that facilitates structure identification and access of antigenic probes. After 15-min fixation at room temperatures a coverglass was placed on top of the preparation. The glass coverslip was removed and the microscope slide- containing fixed spermatogenic cells was used for immunocytochemistry (see below). Cells were immunoreacted with affinity purified PFN3 and PFN4 (working dilution: 1:200) and α-tubulin monoclonal antibody (working dilution 1:100; Sigma-Aldrich, St. Louis, MO), followed by anti-rabbit IgG-conjugated with fluorescein isothiocyanate or anti-mouse IgG conjugated with rhodamine (working dilution 1:200; Jackson Immunoresearch Laboratories, West Grove, PA), respectively. Phalloidin-Texas Red-X was used to detect F-actin according to the manufacturer's protocol (Molecular Probes, Eugene, OR). Specimens were mounted with Vectashield (Vector Laboratories) and examined in a Zeiss Universal phase-contrast/fluorescence microscope equipped with episcopic illumination. Images were recorded using a Magnafire digital CCD camera (Optronics, Goleta CA).

### Generation of 3-dimensional models

Homology models for both PFN3 and PFN4 from mouse and man were generated using the SWISS-MODEL server [50], based on the closest sequence homologues found from the PDB for each protein. For PFN3, the model is based on bovine profilin 1 (PDB entry 1PNE) [51], and for PFN4, the template was Acanthamoeba profilin II (PDB entry 2ACG) [35]. The structures were superimposed with each other and profilins 1 and 2 using the SSM method [52] in Coot [53]. Protein structure figures were generated using Pymol, Dino, and POV-Ray.

## Abbreviations

The abbreviations used are: A: Adenine; BSA: bovine serum albumin; CCD: charge-coupled device; DTT: Dithiothreitol; EDTA: ethylenediaminetetraacetic acid; ELISA: enzyme-linked immunosorbent assay; FITC: fluorescein isothiocyanate; GST: glutathione S-transferase; H: Histidine; HRP: horse reddish peroxidase; L: Leucin; mDia3: mammalian homologue of Drosophila diaphanous, isoform 3; OD: optical density; ON: Over night; PA: phosphatidic acid; PBS: phosphate-buffered saline; PFN: profilin; Pha-Co: phase contrast; pI: isoelectric point; PLP: poly-L-proline; PRD: proline-rich domain; PtdIns(4,5)P_2_: Phosphatidylinositol 4,5-bisphosphate; PtdIns(3)P: Phosphatidylinositol 3-Phosphate; PtdIns(4)P: Phosphatidylinositol 4-Phosphate; SD: single dropout (synthetic minimal medium); T: Thymidin; TBS-T: Tris-buffered saline with Tween-20; VASP: vasodilator-stimulated phosphoprotein; Y2H: Yeast two-hybrid.

## Authors' contributions

MB performed affinity chromatography, yeast-two-hybrid interaction assays, co-immunoprecipitation, Western blot analyses, and immunofluorescence studies. KM performed recombinant protein expression, actin polymerization and protein-lipid overlay assays. PK generated 3-dimensional models and draftet the corresponding part of the manuscript. HCO designed and carried out molecular methods in preparation of yeast-two-hybrid interaction and co-immunoprecipitation assays. AK performed rat immunofluorescence studies, analysed the data, and edited the corresponding part of the manuscript. MR and CK conceived and designed all experimental work, analysed the data, and draftet the manuscript. All authors read and approved the final manuscript.

## Supplementary Material

Additional File 1 **Immunolocalization of PFN4-related protein in spermatids and testicular spermatozoa isolated from human testis**. A1-A3) and B1-B3) show dual labelling and confocal microscopy of human round and elongating spermatids employing indirect PFN4 immunofluorescence (green) and PNA lectin binding (red); nuclei were stained with DAPI (dark blue). A4 and B4 show corresponding phase contrast image. Note PFN4 immunofluorescence in acroplaxome and manchette (high lightened by yellow arrows); spermatocyte shows weak cytoplasmic staining. Scale bars correspond to 5 μm. C1–C3 and D1–D3 show dual labelling and confocal microscopy of human testicular spermatozoa employing indirect PFN4 immunofluorescence (green) and PNA lectin binding (red); nuclei were stained with DAPI (dark blue). C4 and D4 show corresponding phase contrast image. Scale bars correspond to 20 μm and 5 μm, respectively. Click here for file

Additional File 2 **Sequence alignments for homology modeling**. A. Sequence alignment used for the generation of the human PFN3 model. B. Sequence alignment for making the human PFN4 model. Click here for file
