# Supplementary figures and images for: Testis-expressed profilins 3 and 4 show distinct functional characteristics and localize in the acroplaxome-manchette complex in spermatids
Source: BMC Cell Biol. 2009 May 6;10:34. doi: 10.1186/1471-2121-10-34 (PMC2694148; doi:10.1186/1471-2121-10-34)

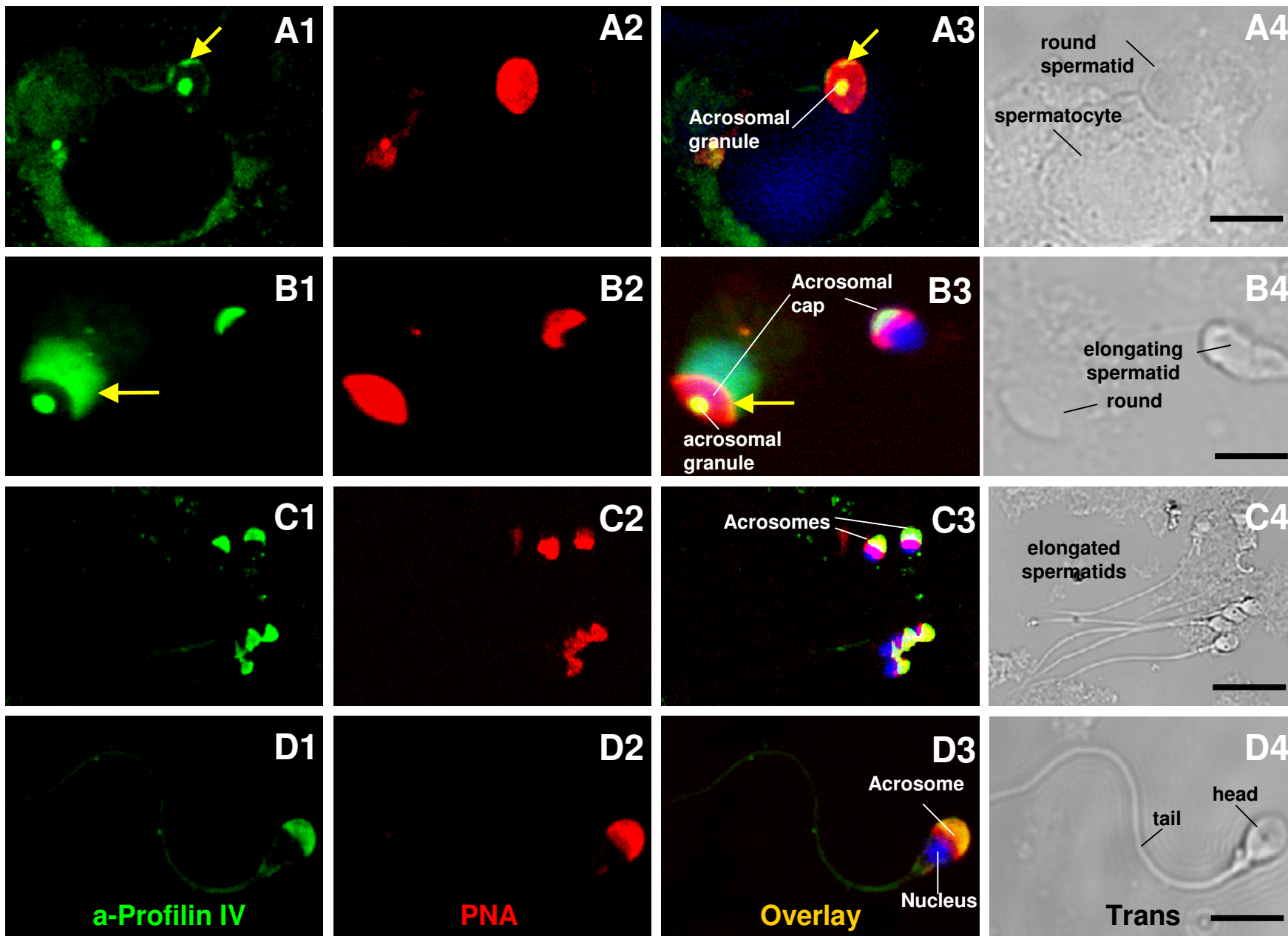

Supplement: Additional File 1 — Immunolocalization of PFN4-related protein in spermatids and testicular spermatozoa isolated from human testis. A1-A3) and B1-B3) show dual labelling and confocal microscopy of human round and elongating spermatids employing indirect PFN4 immunofluorescence (green) and PNA lectin binding (red); nuclei were stained with DAPI (dark blue). A4 and B4 show corresponding phase contrast image. Note PFN4 immunofluorescence in acroplaxome and manchette (high lightened by yellow arrows); spermatocyte shows weak cytoplasmic staining. Scale bars correspond to 5 μm. C1–C3 and D1–D3 show dual labelling and confocal microscopy of human testicular spermatozoa employing indirect PFN4 immunofluorescence (green) and PNA lectin binding (red); nuclei were stained with DAPI (dark blue). C4 and D4 show corresponding phase contrast image. Scale bars correspond to 20 μm and 5 μm, respectively. [file 1471-2121-10-34-S1.pdf]

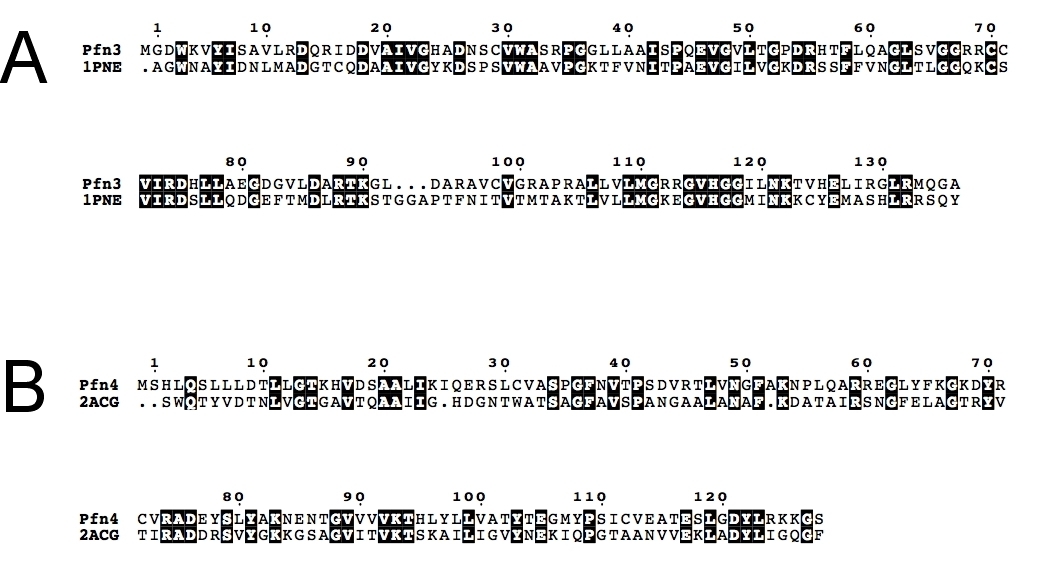

Supplement: Additional File 2 — Sequence alignments for homology modeling. A. Sequence alignment used for the generation of the human PFN3 model. B. Sequence alignment for making the human PFN4 model. [file 1471-2121-10-34-S2.jpeg]
